# Combining Deep Phenotyping of Serum Proteomics and Clinical Data via Machine Learning for COVID-19 Biomarker Discovery

**DOI:** 10.3390/ijms23169161

**Published:** 2022-08-15

**Authors:** Antonio Paolo Beltrami, Maria De Martino, Emiliano Dalla, Matilde Clarissa Malfatti, Federica Caponnetto, Marta Codrich, Daniele Stefanizzi, Martina Fabris, Emanuela Sozio, Federica D’Aurizio, Carlo E. M. Pucillo, Leonardo A. Sechi, Carlo Tascini, Francesco Curcio, Gian Luca Foresti, Claudio Piciarelli, Axel De Nardin, Gianluca Tell, Miriam Isola

**Affiliations:** 1 Department of Medicine (DAME), University of Udine, 33100 Udine, Italy; 2 Academic Hospital of Udine (ASUFC), 33100 Udine, Italy; 3 Department of Mathematics, Informatics and Physics (DMIF), University of Udine, 33100 Udine, Italy

**Keywords:** proximity extension assay, inflammation, cardiometabolic, neurologic disease, COVID-19

## Abstract

The persistence of long-term coronavirus-induced disease 2019 (COVID-19) sequelae demands better insights into its natural history. Therefore, it is crucial to discover the biomarkers of disease outcome to improve clinical practice. In this study, 160 COVID-19 patients were enrolled, of whom 80 had a “non-severe” and 80 had a “severe” outcome. Sera were analyzed by proximity extension assay (PEA) to assess 274 unique proteins associated with inflammation, cardiometabolic, and neurologic diseases. The main clinical and hematochemical data associated with disease outcome were grouped with serological data to form a dataset for the supervised machine learning techniques. We identified nine proteins (i.e., CD200R1, MCP1, MCP3, IL6, LTBP2, MATN3, TRANCE, α2-MRAP, and KIT) that contributed to the correct classification of COVID-19 disease severity when combined with relative neutrophil and lymphocyte counts. By analyzing PEA, clinical and hematochemical data with statistical methods that were able to handle many variables in the presence of a relatively small sample size, we identified nine potential serum biomarkers of a “severe” outcome. Most of these were confirmed by literature data. Importantly, we found three biomarkers associated with central nervous system pathologies and protective factors, which were downregulated in the most severe cases.

## 1. Introduction

Since its first outbreak in Wuhan in 2019, the global pandemic of COVID-19, caused by the severe acute respiratory syndrome coronavirus 2 (SARS-CoV-2), has affected more than 300 million people and caused more than 5.5 million deaths (https://covid19.who.int, accessed on 15 January 2022) [1,2]. From a clinical standpoint, COVID-19 is a challenge due to the heterogeneity of both individual susceptibilities to SARS-CoV-2 infection and disease severity, which ranges from an asymptomatic form to a critical illness with high rates of lethal complications [3,4,5,6].

Part of this heterogeneity could be attributed to the high mutation rates of SARS-CoV-2, a single-stranded enveloped RNA virus. In analogy with other coronaviruses (CoV), with which it shares 79% of sequence identity, the Spike (S) protein of SARS-CoV-2 is required for cell infection via the Angiotensin Converting Enzyme (ACE)-2 receptor [7]. Crucial mutational events, involving the coding sequence of the S protein, enable bat coronavirus to infect human cells and possibly account for the zoonotic transfer [2,8]. Recent studies have demonstrated the high complexity of the SARS-CoV-2 transcriptome owing to numerous recombination events, including the presence of different ORFs bearing fusions, deletions, and/or frameshift mutations [9]. When these events also involve the coding region of the Spike protein, novel, more infectious variants emerge that could escape the immune system, as in the case of Omicron (B.1.1.529) [10].

Another component responsible for the heterogeneity of the clinical presentation could be ascribed to host features. Aside from ethnicity and social determinants, age, sex, smoking habits, and comorbidities, such as diabetes, obesity, cardiovascular and cerebrovascular disease, chronic kidney disease (CKD), chronic obstructive pulmonary disease (COPD), and chronic liver disease and malignancy have been associated with COVID-19 poor outcome [5,11]. Additionally, the genetic background of the individual has been associated both with susceptibility and disease severity. Among the most important elements, we should acknowledge genomic variants affecting the expression of factors critically involved in: viral entrance (e.g., *ACE2*, *Transmembrane Serine Protease 2-TMPRSS-*, *Cathepsin B* and *L*, the blood group antigens A and O), innate antiviral response (e.g., *Oligoadenylate* Synthetase-*OAS-* and *Interferon (IFN) alpha/beta Receptor 2*-*IFNAR2-*), inflammation (e.g., *Dipeptidyl Dipeptidase 9-DPP9-*, *Tyrosine Kinase 2-TYK2-*, *Toll-like Receptor 7-TLR7-*, and *C-C Chemokine Receptor Type 2-CCR2-*), and immunity (e.g., *Human Leukocyte Antigen E-HLA-E-*, *Killer Cell Lectin-like Receptor C2-KLRC2-*) [11,12,13]. Finally, the persistence of memory T cells cross-reactive with other coronaviruses has been evoked as a potentially protective factor against SARS-CoV-2 infection [6].

Critically ill patients are characterized by a hyperinflammatory state associated with endothelitis, hypercoagulability, organ damage, and death [4]. Indeed, a dysregulated immune response is associated with patient outcomes and, in extreme cases, can also trigger multi inflammatory syndrome in children (MISC) [14]. Indeed, literature data show that SARS-CoV-2 infection causes an increased secretion of Interleukin (IL)-1β, Interferon (IFN)-γ, Interferon Gamma-Induced Protein 10 (IP-10), Monocyte Chemoattractant Protein (MCP)-1, IL-4, and IL-10 [15], while disease severity has been associated with an elevation of IL-2, IL-7, Granulocyte Colony-Stimulating Factor (GCSF), Macrophage Inflammatory Protein-1 Alpha (MIP-1A), Tumor Necrosis Factor alpha (TNF-α), IL-10, IP-10, and MCP-1 [15,16,17]. The cardiovascular system is also crucial in dictating the outcome of COVID-19 patients, and cardiac damage markers, such as troponins and brain-type natriuretic peptide (BNP), have been associated with increased mortality [7]. Last, several neurological (e.g., anosmia and ageusia) and psychiatric symptoms have been described in COVID-19 patients, most of which are independent from a direct affection of the central nervous system (CNS) by the virus but are considered to be secondary to the immune reaction [18,19]. CNS involvement is also relevant for the post-acute sequelae of SARS-CoV-2 infection (PASC), a spectrum of symptoms experienced by COVID-19 patients that persist after the resolution of the infection, which is characterized by the high prevalence of neurologic symptoms [19,20]. However, novel studies investigating the evidence of CNS involvement in COVID-19 patients are needed [21].

Identifying new biomarkers associated with disease severity is both crucial to help understand the natural history of COVID-19 and to prospectively identify patients at high risk of developing a critical illness in order to target the allocation of resources, the escalation of care, and inclusion in experimental clinical trials to those who are in most need.

Analytically, there is the need to increase the number of biomolecules assessed per volume of sample to maximize the information obtained and spare biological material [22]. Proximity extension assay (PEA) is a high-throughput technology developed for the high-multiplex analysis of proteins, allowing a successful detection and quantification of several biomarkers [23] by incubating a low amount of the biological sample. To date, some comprehensive studies identifying sera biomarkers correlated with COVID-19 disease severity have been published [24,25,26,27,28,29], but only few of them [25] have employed machine learning techniques to reach a holistic view that summarizes an extensive clinical characterization (including demographic, comorbidity, clinical, and hematochemical data) of the patients with a large number of potential biomarkers in order to identify those that more effectively classify patients with adverse prognosis.

Given these premises, we decided to compare the serum levels of 274 proteins associated with inflammation, cardiometabolic, and neurologic diseases employing PEA analysis in a case series of 160 COVID-19 patients, dichotomized based on COVID-19 severity. Age, comorbidities, and vaccination status significantly differed between the two groups of patients. Analyses were carried out employing machine learning techniques and nine proteins were identified as being able to classify, when combined with relative lymphocyte and neutrophil counts, disease severity. Of note, neurology-associated protein biomarkers were among the strongest predictors of COVID-19 severity.

## 2. Results

### 2.1. Patients’ Characteristics

Between February and September 2021, we enrolled 160 subjects infected by SARS-CoV-2, including 80 patients who were either paucisymptomatic or affected by a mild to moderate form of the disease (here referred to as “non-severe”) and 80 patients who were either affected by a severe to critical COVID-19 at disease onset or who had moderate disease at the onset that worsened and required admission to the ICU (here referred to as “severe”). Demographic, risk factor, and comorbidity data of the enrolled population are summarized in Table 1. Enrolled patients were mainly males, older than 65 years, on average overweight (median BMI 28.2), and had a median Charlson comorbidity index (CCI) of 3. Importantly, having been conducting the enrollment at the beginning of the vaccine campaign, 85% of patients had still not been vaccinated. Although the genotypization of the SARS-CoV-2 strain was conducted only in a minority of patients, available data indicate that they were affected mostly by either the alpha (B.1.1.7) or delta (B.1.167.2) variants of concern (VOC), in consistence with the survey conducted by the Istituto Superiore di Sanità (ISS, Italy) and the data of strain prevalence obtained at our institution during the same timeframe [30,31]. The “severe” patients were characterized by significantly older age, higher CCI, and lower vaccination rate in comparison to the “non-severe” ones.

The clinical outcome of the enrolled patients is summarized in Table 2. As expected, the clinical presentation of the whole case study was heterogeneous, including paucisymptomatic and critical patients, requiring invasive mechanical ventilation (IMV) in the Intensive Care Unit (ICU). The median duration of in-hospital stay was 9 days, and the mean rate of in-hospital death was 56%. The duration of in-hospital stay and the necessity for Continuous Positive Airway Pressure (CPAP) and IMV were significantly higher in the “severe” group of patients. Lastly, while no patient in the “non-severe” group was either admitted to the ICU or died, 51% and 56% of those in the “severe” group met these unfavorable outcomes.

These data indicate that the two subsets of “severe” and “non-severe” patients have distinct, non-overlapping demographic and clinical baseline features with opposing clinical outcomes.

### 2.2. Clinical and Hematochemical Variables Associated with Patient Outcome

**Figure 1:**
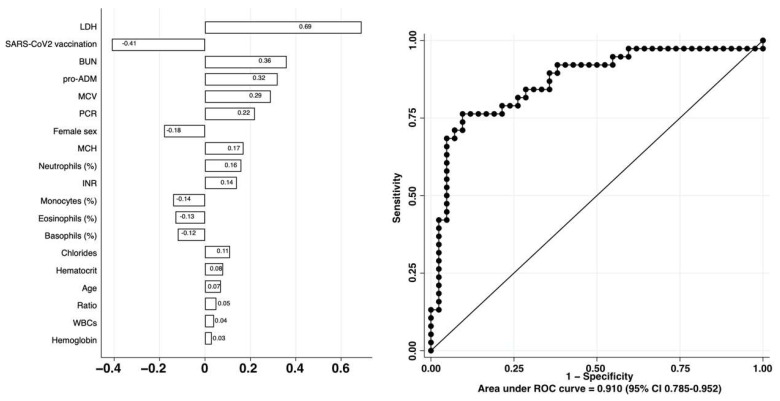
Elastic net logistic regression with hematochemical variables. Receiver Operating Characteristic (ROC), Area Under the Curve (AUC) with its 95% confidence interval (CI) are 0.868 (95% CI 0.785–0.952).

Next, we analyzed 32 different hematochemical variables collected at the time of enrollment (Supplementary Table S2) describing the hematologic, coagulation cascade, liver, kidney, and cardiac compensation of the enrolled patients together with 26 additional demographic and anamnestic variables (Supplementary Table S2). The identification, among the 58 variables, of the strongest classifiers of COVID-19 severity was assessed by employing an elastic net logistic regression approach with cross-validation. As shown in Figure 1, vaccination against SARS-CoV-2 and female sex had a protective effect, while patient age was weakly associated with a worse outcome.

Hematological abnormalities associated with unfavorable outcomes mostly involved the white blood cell (WBC) lineage. Specifically, while the WBC count was weakly associated with a worse outcome, relative neutrophilia, relative monocytopenia, and a relative reduction of basophils and eosinophils were more strongly associated with a worse clinical outcome. The defects also involved the red blood cell lineage, where increased mean corpuscular volume and mean cell hemoglobin were associated with disease severity. These data are consistent with what has already been reported in the literature [32,33]. Concerning the coagulation cascade, we observed that prothrombin time (PT) and activated partial thromboplastin time (aPTT) increases were associated with disease severity. Furthermore, kidney dysfunction (increased blood urea nitrogen—BUN) and electrolyte imbalance were both associated with adverse outcomes in our cohort. Last, we found, in line with literature data, that severe patients are characterized by increased levels of the markers of cell damage (e.g., lactate dehydrogenase—LDH) [34], vascular permeability, stabilization of microcirculation (e.g., mid regional pro-adrenomedullin—MR-ProADM) [35,36], and inflammatory markers (e.g., C reactive protein—CRP) [37]. The model built by employing the above-described parameters showed an excellent ability to classify the patients of the independent dataset, with an area under the curve (AUC) of 0.868 (95% CI 0.785–0.952).

### 2.3. Identification of Serum Proteins Associated with Clinical Outcome

To add new pieces of information concerning the role of the immune, cardiovascular, and nervous systems in COVID-19 pathophysiology, we analyzed patient sera for the expression of 274 different proteins.

**Figure 2:**
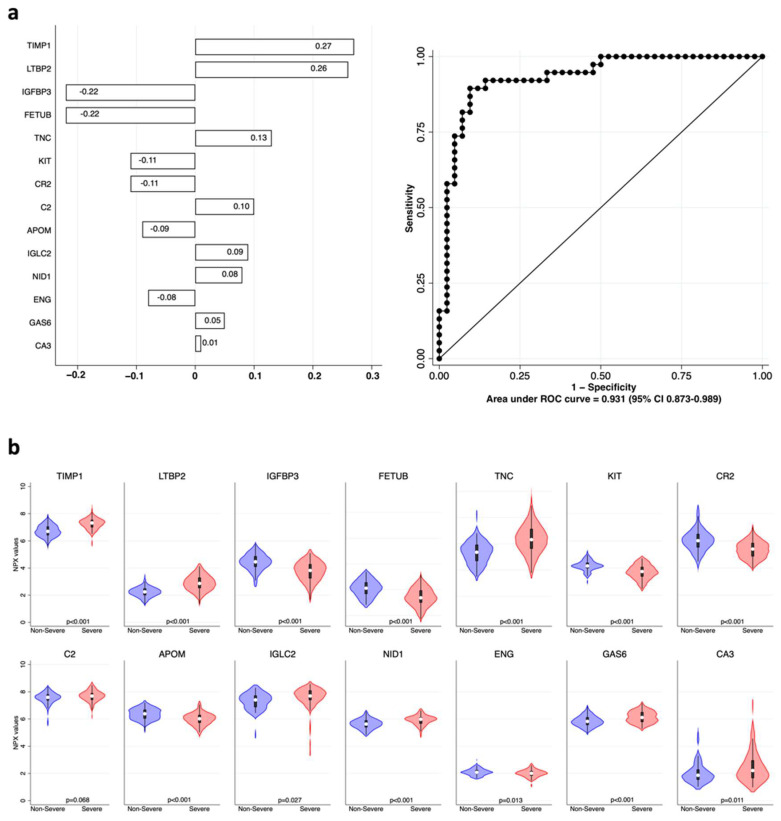
Cardiometabolic biomarkers associated with COVID-19 severity. (**a**) Elastic net logistic regression with cardiometabolic variables. Coefficients of each variable are shown in the left panel as horizontal bars. The right panel shows that the receiver operating characteristic (ROC) and area under the curve (AUC), with its 95% confidence interval (CI) of the model, are 0.931 (95% CI 0.873–0.989). (**b**) Violin plots showing the distribution of the normalized protein expression (NPX) values of each variable included in the model in “non-severe” (blue) vs. “severe” (red) patients; *p* values for each comparison are shown at the bottom of each plot.

First, cardiometabolic-related proteins were analyzed to identify classifiers of disease severity using elastic net logistic regression with cross-validation. Results are shown in Figure 2 and literature data confirming our findings are quoted in Table 3. The complete model containing the 13 markers showed, by ROC analysis, an excellent ability to correctly classify the remaining 80 patients of the dataset, with an AUC of 0.931 (95% CI 0.873–0.989).

**Figure 3:**
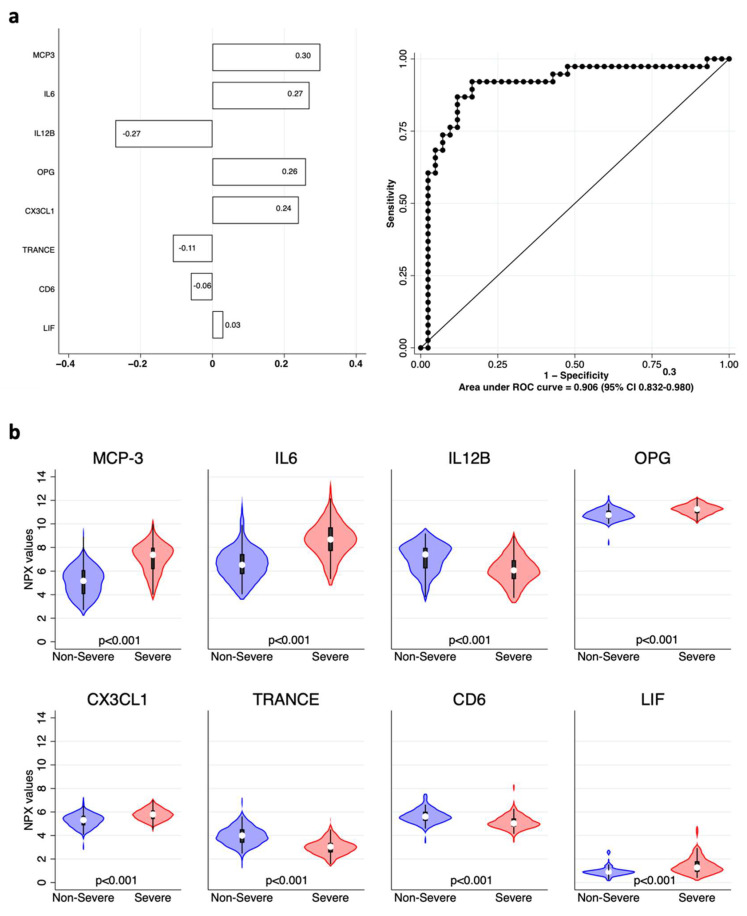
Inflammatory biomarkers associated with COVID-19 severity. (**a**) Elastic net logistic regression with inflammatory variables. Coefficients of each variable are shown in the left panel as horizontal bars. The right panel shows the ROC and AUC of 0.906 (95% CI 0.832–0.980). (**b**) Violin plots showing the distribution of the normalized protein expression (NPX) values of each variable included in the model in “non-severe” (blue) vs. “severe” (red) patients; *p* values for each comparison are shown at the bottom of each plot.

Next, we analyzed inflammatory biomarkers, employing the same approach. Results are shown in Figure 3 and literature data confirming our findings are quoted in Table 3. The model containing the eight markers was able to correctly classify the remaining 80 patients with an AUC of 0.906 (95% CI 0.832–0.980).

**Figure 4:**
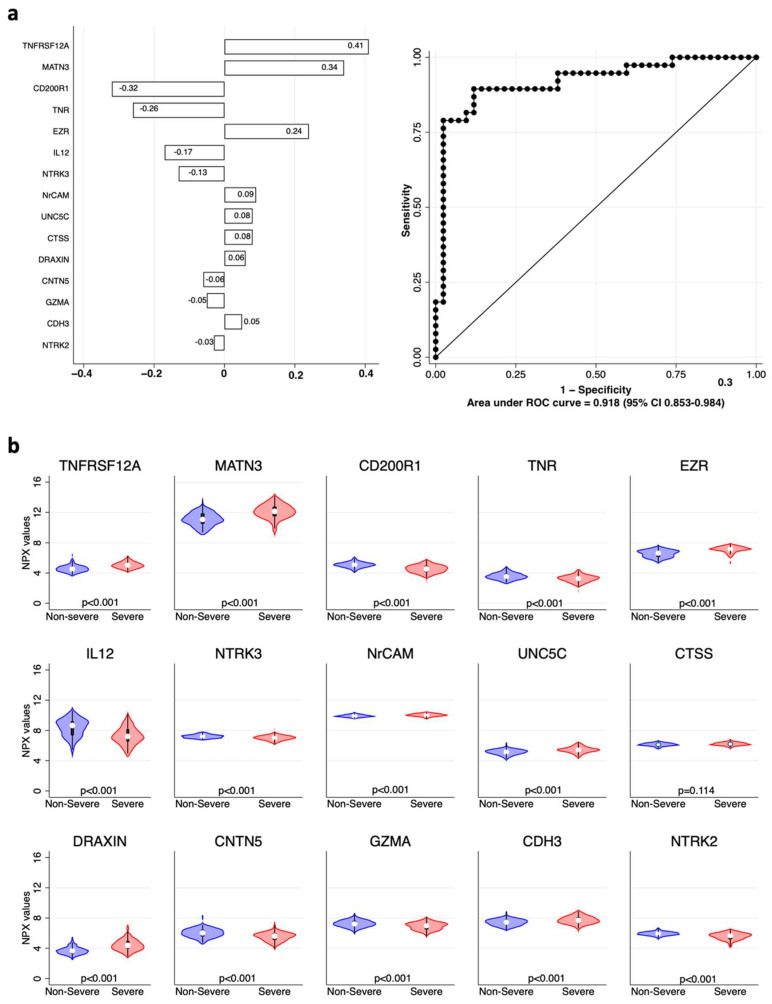
Neurology biomarkers associated with COVID-19 severity. (**a**) Elastic net logistic regression with neurology-related variables. Coefficients of each variable are shown in the left panel as horizontal bars. The right panel shows the ROC and AUC of 0.918 (95% CI 0.853–0.984). (**b**) Violin plots showing the distribution of the normalized protein expression (NPX) values of each variable included in the model in “non-severe” (blue) vs. “severe” (red) patients; *p* values for each comparison are shown at the bottom of each plot.

The last analyzed panel included neurology-related protein biomarkers. Results are shown in Figure 4 and literature data confirming our findings are quoted in Table 3. Again, the complete model had an excellent ability to classify severe cases with an AUC of 0.918 (95% CI 0.853–0.948).

### 2.4. Correlation Discovery Analysis

Last, we aimed to identify the most relevant parameters, able to stratify COVID-19 severity by combining the clinical, demographic, anamnestic, and hematochemical data of each patient with the biomarkers of the three different panels.

**Figure 5:**
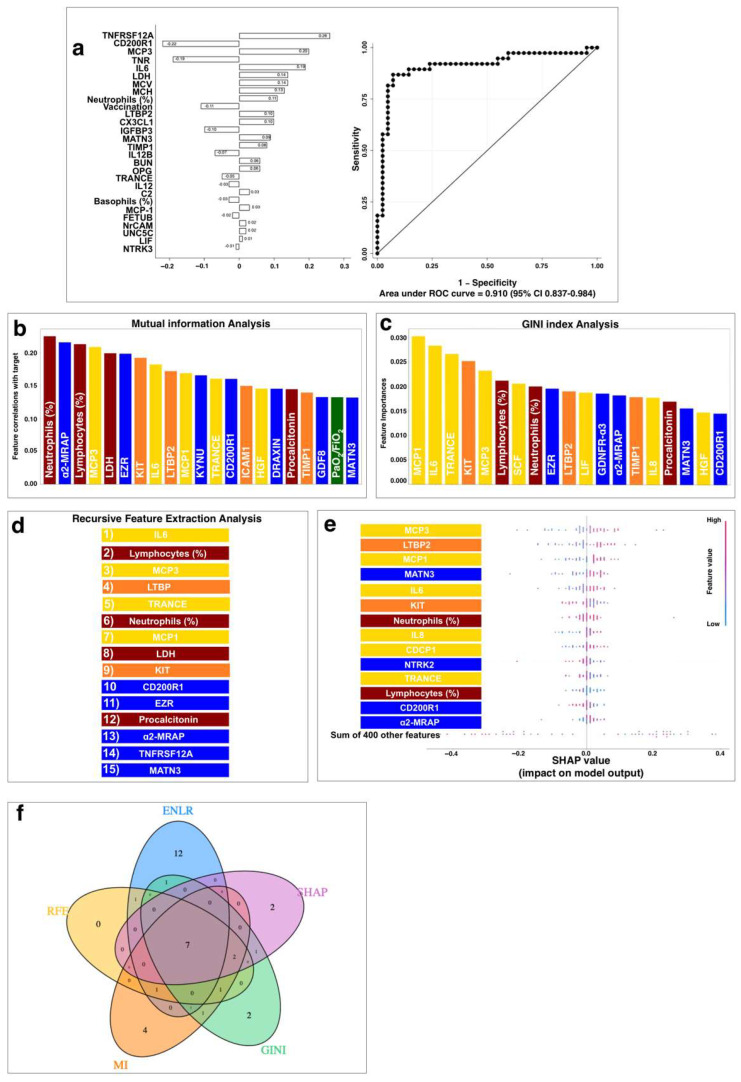
Correlation discovery analysis. (**a**) Elastic net logistic regression with hematochemical, cardiometabolic, inflammatory, and neurology-related variables. ROC and AUC with its 95% confidence interval (CI) are 0.910 (95% CI 0.837–0.984). (**b**) Results of the mutual information analysis. On the *x*-axis, the dataset parameters are provided ranked based on their MI value related to the target variable. (**c**) Ranking of the dataset parameters based on the GINI index values obtained by the adopted random forest mode. (**d**) The ranking of the features obtained through the RFE algorithm. (**e**) The graph was obtained through the SHAP analysis approach. On each row of the graph, the value of the corresponding feature for each instance of the dataset is represented by a dot. The color of the dot indicates how large the value of the feature is in that instance (blue dot: small value, red dot: large value). Furthermore, the position of the dot with respect to the central vertical line indicates whether that feature led the model to classify the patient as a severe case (dot on the right side of the line) or not (dot on the left side). As a clarifying example, we can see that for the MCP3 parameter higher values are correlated with a severe condition for the patient, as the red dots are mostly on the right side of the graph, while the instances in which the MCP3 value is low are often correlated with a non-severe condition for the patient. In (**b**–**e**), the parameters are color-coded to represent their category (red: hematochemical; yellow: inflammatory biomarkers; orange: cardiometabolic biomarkers; blue: neurological biomarkers; green: clinical data). (**f**) Venn diagram of the variables shared between the five models. The eight variables shared by all the models are Neutrophil count, MCP3, IL6, TRANCE, MCP1, CD200R1, MATN3, and LTBP2; KIT and a-MRAP are shared by four models.

For this purpose, we first employed the elastic net logistic regression approach with the cross-validation described previously. Results are shown in Figure 5a and Table 4. The complete model was characterized by an 89.2% sensitivity (95% CI 74.6–97), an 88.4% specificity (95% CI 74.9–96.1), an 86.8% positive predictive value (95% CI 71.9–95.6), a 90.5% negative predictive value (95% CI 77.4–97.3), and 0.776 Youden’s J statistic.

As machine and deep learning techniques are becoming more and more prominent in the medical field [59] with notable examples, especially in automated diagnosis [60] and biomedical images anomaly detection [61], we provided a complementary approach, which relies on four different feature correlation algorithms aiming to highlight the correlation between each of the parameters present in the complete dataset, including the 26 demographic, clinical, and anamnestic parameters; the 32 hematochemical and immunometric parameters together with the plasma proteomics data of 274 different proteins; and the target variable indicating the severity of the each patient’s conditions. Both model-independent and model-dependent approaches were employed.

Results of the model-independent mutual information (MI) analysis [62] are reported in Figure 5b and Table 4. Specifically, MI identified four hematochemical parameters (i.e., relative neutrophil and lymphocyte counts, LDH, and procalcitonin), the clinical parameter PaO_2_/FiO_2_, seven neurology associated markers, five inflammatory markers, and four cardiometabolic factors associated with outcome.

The results obtained by three model-dependent approaches are reported in Figure 5c–e and Table 4. Specifically, GINI index analysis (Figure 5c) identified two hematochemical parameters (i.e., relative neutrophil and lymphocyte counts) together with eight inflammatory markers, five neurology associated markers, and three cardiometabolic factors associated with outcome. Recursive feature extraction analysis (RFE, Figure 5d) identified four hematochemical parameters (i.e., relative neutrophil and lymphocyte counts, LDH, and procalcitonin), combined with five neurology-associated markers, and two cardiometabolic factors associated with outcome. Last, according to the Shapley additive explanations (SHAP, Figure 5e) analysis, two hematochemical parameters (i.e., relative neutrophil and lymphocyte counts), combined with six inflammatory markers, four neurology-associated markers, and two cardiometabolic factors were correlated with the outcome. Intriguingly, no clinical parameter emerged from model-dependent approaches.

Finally, we assessed which variables were associated with disease severity that were shared in all the above-mentioned approaches (Figure 5f and Table 4). The relative Neutrophil count, together with the inflammatory markers MCP3 [50], IL6 [16,51], TRANCE [63], and MCP1 [24]; the neurology-associated markers CD200R1 [54] and MATN3 [54]; and the cardiometabolic marker LTBP2 [39] emerged in every analysis conducted. Lymphocyte count, KIT, and α2-MRAP [54] were shared by the four different feature correlation algorithms.

### 2.5. Functional Enrichment Analysis of Proteins Associated with the Disease Outcome

**Figure 6:**
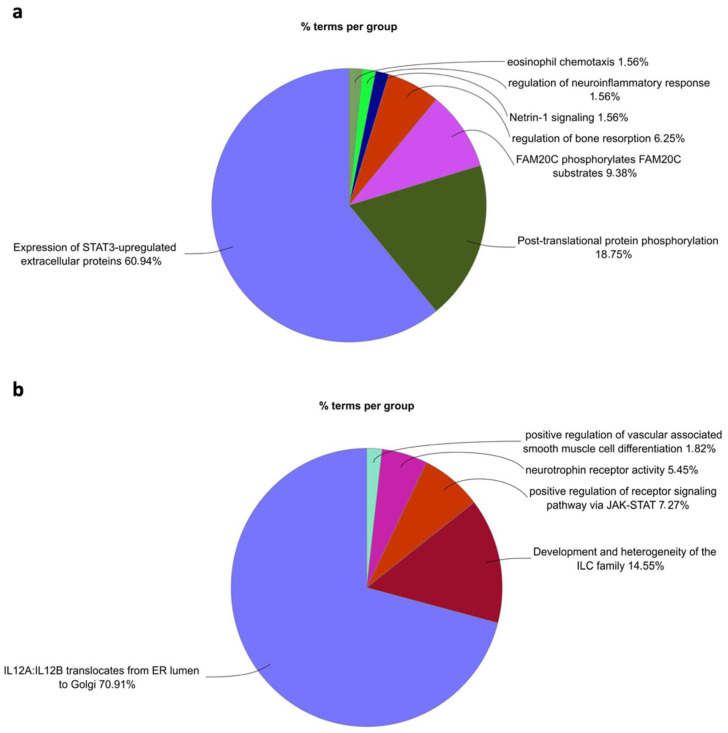
Functional enrichment analysis of proteins associated with a “severe” outcome. Pie charts representing the most enriched functional terms (padj ≤ 0.05) related to proteins whose coefficients were positively (**a**) and negatively (**b**) associated with the “severe” class. The Cytoscape [65] app ClueGO [66] was used to query the following functional databases: GO (BiologicalProcess and ImmuneSystemProcess), KEGG, REACTOME, CLINVAR, WikiPathways, and CORUM-3.0.

To associate a functional role to the proteins discriminating the “severe” patients from the “non-severe” ones, potential biomarkers emerging from the cardiometabolic, inflammation, and neurology panels were subjected to the ClueGO functional enrichment analysis. The elastic net logistic regression model built by employing the proteins profiled with the three panels together with clinical, demographic, and hematochemical data (Figure 5a) included, except for MCP1, all the predictors emerging from the analyses conducted by separately employing every single panel. Therefore, we decided to group the proteins emerging from the first three models (Figure 2, Figure 3 and Figure 4) and to conduct the functional analysis, distinguishing those positively associated with disease severity from those negatively associated with a “severe” COVID-19. As shown in Figure 6a, most of the proteins positively associated with disease severity pertained to the functional terms “expression of STAT-3 upregulated extracellular proteins”, followed by “Post-translational protein phosphorylation”, “FAM20C phosphorylates FAM20C substrates”, “Regulation of bone resorptions”, “Netrin-1 signaling”, “Regulation of neuroinflammatory response”, and “Eosinophil chemotaxis”. Employing the same dataset to query the Molecular Signature Database (MSigDB) [64], we observed that the upregulated genes were associated with the hallmarks: “inflammatory response”, “allograft rejection”, and “epithelial to mesenchymal transition”. Consistently, several mesenchymal cell types emerged.

Conversely, proteins negatively associated with disease severity (Figure 6b) are enriched in the functional terms: “IL12A-IL12B translocate from ER lumen to Golgi”, “Development and heterogeneity of the ILC family”, “Positive regulation of receptor signaling via JAK-STAT”, “Neurotrophin receptor activity”, and “Positive regulation of vascular associated smooth muscle cell differentiation”. The MSigDB analysis of the terms inversely associated with severity identified several lymphocyte-related terms.

## 3. Discussion

Here we present the results of an extensive analysis of the serum proteome of 160 adult patients affected by the SARS-CoV-2 infection during a phase of the COVID-19 pandemic (i.e., February–September 2021) dominated by the VOC Alpha and Delta [30,31]. In this study we extensively used PEA, an emerging technology that can simultaneously measure, with high sensitivity and specificity, 92 proteins across 96 samples, using only 1 µL of serum [67]. Given the involvement of both inflammation [3] and cardiac injury (triggered both by direct [68] and indirect mechanisms [69]) in the pathophysiology of COVID-19, two commercial panels containing 92 “inflammation” and “cardiometabolic” biomarkers were employed. Since a large number of neurological manifestations (e.g., headache, confusion, neuroinflammatory, and cerebrovascular disease) have been described to occur in the acute and chronic phase of COVID-19 [20,70], we additionally employed a commercial panel containing 92 “neurology” biomarkers.

Furthermore, we employed machine learning (ML) techniques to identify the strongest predictors of disease severity, by aggregating 26 clinical, demographic, and anamnestic parameters (including the major risk factors for severe COVID-19 and the vaccination status of the enrolled patients) and 32 hematochemical and immunometric parameters (comprising markers of inflammation—CRP [71], myocardial injury—cardiac Troponin [72], severe bacterial infection—procalcitonin [72], and emerging markers of sepsis—MR-proADM [72], which have been associated with COVID-19 outcome) together with the plasma proteomics data of 274 different proteins. Although several works aiming at identifying the biomarkers of severe COVID-19 have been published [24,25,26,27,29,45], some of which have also taken advantage of PEA technology, we believe that our work has two major strengths: (1) this is one of the largest cohorts of adult patients profiled so far, after [25,27,29], and (2) ML approaches were employed to explore which of the clinical, hematochemical, and biomarker parameters characterizing patients at admission are able to stratify outcomes.

To analyze our database, we first employed an elastic net logistic approach with cross-validation, a method that performs well even in the case of highly correlated predictors and in the presence of many variables over a smaller sample size. We additionally employed four different ML approaches, both model-independent and model-dependent. The main feature of model agnostic approaches is that they do not rely on a ML model to identify the correlation between the dataset parameters, but they work only on the data itself. The only model-independent approach we selected was the one based on the correlation identification through mutual information (MI) analysis [62]. Concerning model-dependent approaches, they rely on a ML model to infer the correlation between each parameter and the target variable. Here, we specifically selected RFE analysis, feature selection through GINI Index, and SHAP analysis. Each of these provides a higher degree of descriptiveness, which allowed us to gain a deeper understanding of the highlighted correlations. Since these approaches rely on a ML model, the quality of the parameter correlation predictions highly depends on the performance of the latter for the task at hand, in this case, the classification of patients based on the severity of their conditions. The model we selected for our analysis is a random forest [73], consisting of 200 decision trees, while the metrics we adopted to assess its performance during the classification task are, as for the previously described statistical approaches, the ROC–AUC calculated by employing a 10-fold-cross-validation process on the dataset. The obtained result is a ROC–AUC of 0.942 (95% CI 0.935–0.950), which allowed us to proceed with further correlation analysis with confidence.

To identify the variables most strongly associated with COVID-19 severity, we finally compared the results of the five different approaches and observed that the relative neutrophil and lymphocyte count, together with the inflammatory markers MCP3 [50], IL6 [16], TRANCE [63], and MCP1 [24]; the neurology-associated markers CD200R1 [54], α2-MRAP [54], and MATN3 [54]; and the cardiometabolic markers KIT and LTBP2 [39] emerged in at least four of the analyses conducted.

From a pathophysiological standpoint, the dysfunction of both the innate and adaptive immune responses characterize patients with severe COVID-19 [74,75]. Although increased neutrophils and neutrophil-to-lymphocyte ratio have been described in cases of severe COVID-19 [38,76], the active role of neutrophils in COVID-19 pathophysiology has been debated [77]. However, some pieces of evidence support their importance; SARS-CoV-2 can directly induce the release of neutrophil extracellular traps (NETs) from donor-derived neutrophils [78]. These web-like chromatin structures have a role in viral clearance, but excessive NET levels exacerbate inflammation in acute respiratory distress syndrome and have been associated with COVID-19 complications, ranging from thrombosis to CNS involvement [77]. Granulocytes and monocytes activated by a continuous and weak signal (e.g., cytokines) have an immature phenotype and produce immunosuppressive and anti-inflammatory factors [79]. Indeed, the presence of immature neutrophils with reduced functional properties is associated with COVID-19 severity [38,76]. Consistently, most of the patients with severe outcome had microbial superinfections [76], a finding consistent with the recurrence of procalcitonin variable in most of the ML models in our dataset. The profound alterations of the hematopoietic stem cell maturation are observed in COVID-19 patients (i.e., the expansion of the “granulocyte-monocyte progenitor” and the “erythroid progenitor” cell pools, at the expense of the lymphoid cell pool) [80]. In line with this, the emergence of monocytes expressing low levels of HLA-DR and anti-inflammatory molecules, together with neutrophils expressing the immune checkpoint protein PD-L1 [81] coupled with the exhaustion and anergy of T cells, are all hallmarks of severe COVID-19 [82]. Consistently, the relative Lymphocyte count was identified to be associated with disease severity by all the feature correlation algorithms we employed. Moreover, elastic net logistic regression analysis showed an inverse relationship between CR2 plasma levels and disease severity. CR2 (also known as CD21) is present on B lymphocytes, where it is required for their activation. CD21 shedding follows B lymphocyte activation [83], thus a soluble form of CD21 can be found in plasma, reflecting the activity of B-cells [84]. Our results therefore suggest that COVID-19 severity is higher in those patients that show a lower B-cell response. To the best of our knowledge, this association has not been described in the literature yet.

Concerning inflammatory biomarkers associated with disease severity, a hyperinflammatory response with high levels of interferons, cytokines, and chemokines characterizes patients with bad prognosis [15,85]. In line with this, in our cohort, IL6, MCP1, and MCP3 emerged among the strongest independent predictors of disease severity. More complex is the involvement of TRANCE (alias RANKL) in modulating COVID-19 severity. Indeed, this cytokine and its soluble decoy receptor OPG may play a relevant role in the context of acquired immunity [86]. Although these are the main regulators of bone metabolism, TRANCE also enhances the immune response by promoting dendritic cell viability and function [86]. Indeed, the stimulation of dendritic cells with TRANCE triggers an anti-viral CD8^+^ T cell cytotoxic response [87]. Therefore, it is not surprising that we and other authors found TRANCE levels to be inversely associated with disease severity [56].

One of the most innovative findings of our work is that three biomarkers that are also altered in neurological diseases (CD200R1, α2-MRAP, and MATN3) are among the strongest predictors of a negative outcome in the complete model. This finding is supported by the identification, through the functional enrichment analysis of proteins associated with the disease outcome and with “Regulation of neuroinflammatory response” and “neurotrophin receptor activity” terms. Intriguingly, several neurological and psychiatric symptoms have been described in COVID-19 patients, most of which are independent of a direct affection of the central nervous system (CNS) by the virus but are considered to be secondary to the immune reaction [18]. Consistently, we observed an inverse relationship between CD200R1 serum levels and COVID-19 severity. CD200R1 is the receptor for CD200 and has been involved in the inhibition of neuroinflammatory pathways in aging, stroke, and multiple sclerosis [88]. CD200R is also expressed by cells of innate immunity (e.g., monocytes and neutrophils) and regulates their function and maturation [89]. Importantly, the expression of CD200R1 on infiltrating lymphocytes dictates the survival of mice exposed to brain injury by regulating the post-stroke immune suppression and vulnerability of animals to superinfections [90]. Interestingly, the direct injection of the S1 subunit of the SARS-CoV-2 Spike protein in the brain of mice induces neuroinflammation and, after an initial increase, reduces the levels of *Cd200r1* under the level of control animals [19]. Concerning the other neurological markers positively associated with a negative outcome, α2-MRAP (also known as LDL receptor-related protein associated protein -1 or LRPAP1) is involved in the trafficking of LDL receptor family members. Specifically, it regulates the amount of LRP that is expressed in the liver and the brain. LRP binds ApoE and α2-macroglobulin, a protein involved in the clearance of β-amyloid (Aβ). Consistently, LRP-1 is involved in the export of Aβ from the brain [91], while LRPAP1 gene polymorphisms are associated with late-onset Alzheimer’s disease [92]. Importantly, viral infections can drive Aβ formation or deposition, providing a possible link between COVID-19, neuroinflammation, and neurodegeneration [93]. Although recent literature data confirms the association of MATN3 with COVID-19 severity [54], its pathophysiological role is not clear. An additional neurology associated marker that emerged only from the elastic net logistic regression analysis and has not been reported in the literature yet is Tenascin R (TNR). This is a member of the tenascin family of extracellular proteins that is specifically expressed in the CNS. Although another proinflammatory member of this family (i.e., Tenascin C) has been identified in the exosomes of COVID-19 patients [42], no data on Tenascin R could be found in COVID-19-related literature. TNR is part of the highly specialized perineuronal networks of the extracellular matrix, which repel adjacent dendrites and axons to maintain the established synaptic networks [94]. Reduced TNR levels have been described in the cerebro spinal fluid of patients affected by amyotrophic lateral sclerosis (ALS), reflecting the loss of immunoreactivity in the spinal cord of ALS patients. This finding suggests that the control of TNR levels is important to prevent CNS abnormalities [95]. These data are consistent with ours, since patients affected by severe COVID-19 have significantly reduced TNR levels.

Concerning the cardiometabolic biomarker panel, while LTBP2 was higher in patients with a “severe” outcome, KIT showed the opposite behavior. LTBP2 expression increases in response to myocardial stressors (e.g., pressure overload [96] or isoproterenol [97]) and has been associated with both cardiac [96] and pulmonary fibrosis [39]. Consistently, in patients with COVID-19, LTBP2 levels are related to pulmonary fibrosis [39] and are associated with disease severity [98]. In adulthood, the KIT receptor and its ligand SCF are mainly expressed by stem cells and mastocytes and regulate their function [99]. Intriguingly, while hematopoietic stem cells express low levels of ACE2, this receptor, which is involved in SARS-CoV-2 virus entry, is upregulated during erythroid differentiation and parallels KIT expression. Consistently, erythroid progenitors are susceptible to coronavirus infection and possibly account for the increased circulation of nucleated red blood cells following SARS-CoV-2 infection [33]. Regarding mastocytes, lung biopsies have shown an abundant infiltrate of KIT^+^ cells in COVID-19 patients and SARS-CoV-2-infected African green monkeys, suggesting the early recruitment of mastocyte progenitors to the alveolar septa. This morphologic finding was also coupled with interstitial and alveolar edema, suggesting a relevant pathophysiological role for this cell type [100,101]. How these alterations relate to our findings could not be predicted at this time. However, to the best of our knowledge, the serum levels of KIT have not yet been associated with COVID-19 patient outcomes.

By combining a novel high-throughput proteomics approach with ML techniques in a patient population deeply characterized from the clinical standpoint, we identified potential serum biomarkers of a “severe” outcome of COVID-19-affected patients. Our study was limited by its retrospective nature and the limited number of enrolled patients. Although we did not have a validation cohort and we could not find any open access datasets containing the same clinical and hematochemical information we employed, we must underline that most of the biomarkers that emerged from our analysis are confirmed by many, unrelated authors in the literature, further supporting the solidity of our approach. However, to our knowledge, this is the one of the largest studies that used extensive PEA analysis applied to such a large cohort (*n* = 160) of COVID-19-affected patients. One of the major novelties of this work is the demonstration that the markers of neurological disease are among the strongest predictors of COVID-19 severity, when combined with relevant laboratory data, such as lymphocyte and neutrophil counts. Our findings are hypothesis-generating and stimulate studies aimed at understanding the pathophysiological role of neuroinflammation in the longer-term outcome of COVID-19 patients.

## 4. Materials and Methods

### 4.1. Examined Cohort

#### 4.1.1. Patient Enrollment and Ethics

The study, authorized by the Regional Ethic Committee (2020-Os-033; Em. Sost. N. 1 versione 1 data 16 August 2021), was conducted according to the declaration of Helsinki and signed informed consent was collected from enrolled patients. Inclusion criteria were age > 18 years and nasopharyngeal swab positivity for the SARS-CoV-2 genome.

#### 4.1.2. Study Design

This work aims at identifying serum proteins discriminating “non-severe” from “severe” COVID-19 patients, in analogy with recently published works [27]. We classified as “severe”, on top of severe and critical patients according to the WHO classification [102], also those patients presenting a moderate disease at onset that worsened over time, requiring admission to the intensive care unit at a later stage.

#### 4.1.3. Clinical Data

Relevant patient data were collected and extracted by a team of physicians (E.S., C.T.) from the hospital electronic health records (INSIEL, Trieste, Italy), pseudonymized, and recorded on a cloud-based clinical data management platform (Castor, The Netherlands and the USA). Immunocompromised patients were defined as patients with permanent dysfunction of the immune response resulting from immunosuppressive medication or comorbidities, such as AIDS, or malignancies that cause neutropenia (neutrophil counts ≤ 500 cells/mm^3^). Patients with “renal impairment” were defined as having kidney damage or glomerular filtration rate (GFR) < 60 mL/min/1.73 m^2^ for 3 months or more, irrespective of the cause.

### 4.2. Samples

#### 4.2.1. Blood Sampling and Serum Storage

Blood samples, drawn within 1 day (median value) of hospital admission, were collected into a 5 mL serum tube with a clot activator and gel separator (Vacuette, Greiner Bio-One, Nürtingen, Germany), immediately sent to the core laboratory to be processed, and kept at −80 °C until used.

#### 4.2.2. Proximity Extension Assay (PEA)

Sera were analyzed for 92 inflammation-related protein biomarkers (Olink Target 96 Inflammation), 92 cardiometabolic-related (Olink Target 96 Cardiometabolic), and 92 neurology-related protein biomarkers (Olink Target 96 Neurology) employing the proximity extension assay (PEA) technique of Olink^®^ Proteomics (Uppsala, Sweden) [23]. The quality control of the analyzed samples, data normalization, and the quantification of protein levels was performed with the Olink Analyze R package [103], using NPX data files generated from Olink NPX Manager as input. Levels of proteins were described as normalized protein expression (NPX), consisting of data normalization to the extension control (known standard), log2-transformation, and level adjustment using the plate control (plasma sample). We performed exploratory analysis using the ‘olink_qc_plot’ and ‘olink_pca_plot’ functions, generating QC and PCA plots that allowed us to easily identify missing and/or problematic samples.

#### 4.2.3. Statistical Analyses

Descriptive statistics for categorical variables are presented as number (percent) and for continuous variables as mean ± standard deviation (SD) or median (interquartile range (IQR)). Normality was assessed using Shapiro–Wilk test. Comparisons between categorical variables were performed using the Chi-square or Fisher’s exact test, as appropriate. Comparisons between continuous variables were performed using the *t*-test or Mann–Whitney U-test, as appropriate.

An elastic net logistic regression algorithm was used to build different models to predict a “non-severe” or “severe” outcome [104,105]. The elastic net logistic regression is a regularization model that combines, through a linear combination of LASSO and Ridge methods, both L_1_ and L_2_ penalties. This model performs a variable selection, forming a subset of predictors, each one matched with a regression coefficient. Variable importance is ordered using the absolute value of the regression coefficient, a higher value showing a bigger contribution to the model. The dataset was split into testing and training set with a 1:1 proportion. A 10-fold cross-validation was applied to the training set to tune the hyper-parameters λ, determining the amount of shrinkage and α, and explaining the presence of L_1_ and L_2_ penalties. The model was trained for the clinical and hematochemical data, inflammatory, cardiometabolic, and neurological PEA panels separately and jointly. The performance of these model was evaluated on the testing set using the receiver operating curve (ROC) and the area under the curve (AUC), with its 95% confidence interval, compared using Long’s test. Analyses were performed using STATA 17. Table S3 provides hyperparameter and model validation information.

#### 4.2.4. Correlation Discovery Analysis

All the algorithms employed for the correlation discovery analysis have been implemented in Python 3.7.9 by relying on two main tools, the scikit-learn library [106] and the SHAP package [107]. The former was adopted for the development of the random forest model as well as for the recurrent feature extraction, mutual information, and GINI Index approach, while the latter was employed to implement the SHAP component of the analysis.

The only model-independent approach is MI [26]. MI between two random variables is a non-negative value, which measures the dependency between the variables. It is equal to zero if and only if two random variables are independent, and higher values mean higher dependency. For this reason, a higher MI value indicates a higher correlation between the selected parameter and the target variable. Concerning model-dependent approaches:

1. *Recursive feature extraction (RFE) analysis [108]:* an algorithm that at each step removes the features that have the lowest correlation with the target variable for the considered task and then retrains the model on the remaining parameters. The features can then be ranked based on the removal order, where the last feature to be removed is placed in 1st position regarding importance;
2. *Feature selection through GINI Index [73]:* this approach is very similar to the already presented MI one. The main difference is represented by the metric adopted, which in this case is the GINI Index, a measure that represents the amount of information with which each feature contributes to determining the final output of the model. Compared to the RFE algorithm it not only provides a ranking of the features but also a measure of correlation for each;
3. *SHAP (Shapley additive explanations) analysis [107]:* it is the most descriptive of the proposed approaches, as it not only allows the measure of how much a certain feature contributes to the outcome to be obtained but also to which of the outcomes the feature leads the model towards.

The data used for the analysis were kept consistent with the ones described in the previous sections to provide a meaningful comparison between the different approaches. For the data preprocessing, we followed a three-step pipeline. The first step consisted of the removal of those features which, instead of being a predictor of the patient outcome were depending on it (e.g., patient death, hospitalization of the patient in the Intensive Care Unit). Following this first step, we relied on a data imputing procedure to introduce a placeholder where the value for the corresponding feature was missing. Finally, we standardized the data to avoid the results of the analysis being biased towards the features characterized by larger-scale values.

#### 4.2.5. Functional Enrichment Analysis

To characterize the functional role of the most interesting, clinically-relevant identified biomarkers, the Cytoscape app ClueGO was used to query the following functional databases: GO (BiologicalProcess and ImmuneSystemProcess), KEGG, REACTOME, CLINVAR, WikiPathways, and CORUM-3.0. A two-sided hypergeometric test (corrected using the Benjamini–Hochberg method to control the false discovery rate, adjusted *p* ≤ 0.05) was used to determine the probability that each functional term was assigned to the gene sets due to chance alone.

## Figures and Tables

**Table 1:** Summary of baseline characteristics of enrolled patients. Baseline demographic, clinical features (comorbidities), and vaccination status of the enrolled patients (*n* = 160). Patients were stratified according to the severity of the disease into a “non-severe” group and a “severe” one. Data are presented as either percentage or median and interquartile range (IQR). Results of the comparison between “non-severe” vs. “severe” patients are shown in the right column (*p*-value). Significant results are shown in bold.

	Total (*N* = 160)	Non-Severe (*N* = 80)	Severe (*N* = 80)	*p*-Value
**Gender, *n* (%)**				0.251
**Female**	59 (36.9)	33 (41.2)	26 (32.5)	
**Male**	101 (63.1)	47 (58.8)	54 (67.5)	
**Age, years, median (IQR)**	67 (56–76)	61 (50–74)	70 (63–77)	**0.001**
**BMI, median (IQR)**	28.2 (24.9–31.2)	27.8 (24.9–31.1)	28.5 (25.5–31.5)	0.541
**Smoking habit, *n*/*N* (%)**				0.411
**Non-smoker**	79/107 (73.8)	41/53 (77.4)	38/54 (70.4)	
**Smoker**	28/107 (26.2)	12/53 (22.6)	16/54 (29.6)	
**Charlson comorbidity index, median (IQR)**	3 (1–5)	2 (1–5)	4 (2–5.5)	**0.007**
**Comorbidities, *n*/*N* (%)**				
**Hypertension**	79/155 (51.0)	39/76 (51.3)	40/79 (50.6)	0.932
**Obesity**	64/134 (47.8)	30/66 (45.4)	34/68 (50.0)	0.598
**Diabetes**	31/155 (20.0)	13/76 (17.1)	18/79 (22.8)	0.377
**COPD**	16/156 (10.3)	5/77 (6.5)	11/79 (13.9)	0.126
**Cardiovascular disease**	57/156 (36.5)	27/77 (35.1)	30/79 (38.0)	0.706
**Liver disease**	5/155 (3.2)	1/76 (1.3)	4/79 (5.1)	0.187
**Renal impairment**	13/156 (8.3)	6/77 (7.8)	7/79 (8.9)	0.809
**Immunocompromised**	14/147 (9.5)	6/67 (9.0)	8/80 (10.0)	0.830
**COVID-19 vaccination, *n* (%)**	24 (15.0)	20 (25.0)	4 (5.0)	**<0.001**

**Table 2:** Summary of clinical characteristics of enrolled patients. Stratification of the enrolled patients according to the disease severity, necessity for invasive and non-invasive ventilation, admission to the intensive care unit, the duration of hospitalization, death rate, and the duration of infection. Data are presented as either percentage or median and interquartile range. Results of the comparison between non-severe vs. severe patients are shown in the right column (*p*-value). Significant results are shown in bold.

	Total (*N* = 160)	Non-Severe (*N* = 80)	Severe (*N* = 80)	*p*-Value
**Acute COVID-19 severity, *n* (%)**				**<0.001**
**Paucisymptomatic**	16 (10.0)	16 (20.0)	0 (0.0)	
**Mild**	55 (34.4)	55 (68.7)	0 (0.0)	
**Moderate**	14 (8.7)	9 (11.3)	5 (6.3)	
**Severe**	71 (44.4)	0 (0.0)	71 (88.7)	
**Critical**	4 (2.5)	0 (0.0)	4 (5.0)	
**CPAP, *n* (%)**	69 (43.1)	1 (1.2)	68 (85.0)	**<0.001**
**Invasive mechanical ventilation (IMV), *n* (%)**	45 (28.1)	0 (0.0)	45 (56.2)	**<0.001**
**Length of in-hospital stay, days, median (IQR)**	9 (5–15.5)	5.5 (4–9)	14 (9–19)	**<0.001**
**ICU, *n* (%)**	51 (31.9)	0 (0.0)	51 (63.7)	**<0.001**
**In-hospital death, *n* (%)**	56 (35.0)	0 (0.0)	56 (70.0)	**<0.001**
**Duration of nasal swab positivity days, median (IQR)**	19 (14–24)	17 (12–20)	21.5 (17.5–29.5)	**0.008**

**Table 3:** Summary of elastic net logistic regression analyses of biomarkers associated with disease severity.

	Short Name	Biomarker Category	Association with Outcome	Literature Data
**Tissue inhibitor of metalloprotease 1**	TIMP-1	Cardio	Direct	[38]
**Latent-Transforming Growth Factor Beta-Binding Protein 2**	LTBP2	Cardio	Direct	[39]
**Insulin-like Growth Factor Binding Protein 3**	IGFBP3	Cardio	Inverse	[40]
**Fetuin B**	FETUB	Cardio	Inverse	[41]
**Tenascin-C**	TNC	Cardio	Direct	[42]
**KIT proto-oncogene receptor tyrosine kinase**	KIT	Cardio	Inverse	
**Complement C3d Receptor 2**	CR2	Cardio	Inverse	
**Complement component 2**	C2	Cardio	Direct	[43]
**Apolipoprotein M**	APOM	Cardio	Inverse	[44]
**Immunoglobulin Lambda Constant 2**	IGLC2	Cardio	Direct	[45]
**Nidogen-1**	NID1	Cardio	Direct	[46]
**Endoglin**	ENG	Cardio	Inverse	[47]
**Growth Arrest Specific 6**	GAS6	Cardio	Direct	[48]
**Carbonic Anydrase**	CA3	Cardio	Direct	[49]
**Monocyte Chemoattractant Protein 3**	MCP3	Inflammatory	Direct	[50]
**Interleukin 6**	IL6	Inflammatory	Direct	[16,51]
**Interleukin 12B**	IL12B	Inflammatory	Inverse	[52]
**Osteoprotegerin**	OPG	Inflammatory	Direct	[53,54]
**C-X3-C Motif Chemokine Ligand 1**	CX3CL1	Inflammatory	Direct	[54]
**Tumor necrosis factor ligand superfamily member 11**	TNFSF11 or TRANCE, RANKL, OPGL	Inflammatory	Inverse	[54]
**T-cell differentiation antigen CD6**	CD6	Inflammatory	Inverse	[54]
**Leukemia Inhibitory Factor**	LIF	Inflammatory	Direct	[55]
**Tumor Necrosis Factor Receptor Superfamily member 12A**	TNFRSF12A	Neurology	Direct	[56]
**Matrilin 3**	MATN3	Neurology	Direct	[54]
**CD200 Receptor 1**	CD200R1	Neurology	Inverse	[54,57]
**Tenascin R**	TNR	Neurology	Inverse	
**Ezrin**	EZR	Neurology	Direct	[54]
**Interleukin 12**	IL12	Neurology	Inverse	[52]
**Neurotrophic Receptor Tyrosine Kinase 3**	NTRK3	Neurology	Inverse	[54]
**Neuronal Cell Adhesion Molecule**	NrCAM	Neurology	Direct	[58]
**Netrin receptor**	UNC5C	Neurology	Direct	[54] †
**Cathepsin S**	CTSS	Neurology	Direct	[54]
**Dorsal Inhibitory Axon Guidance Protein**	DRAXIN	Neurology	Direct	[54]
**Contactin 5**	CNTN5	Neurology	Inverse	[54]
**Granzyme A**	GZMA	Neurology	Inverse *	[54]
**Cadherin 3**	CDH3	Neurology	Direct	[54] †
**Neurotrophic Receptor Tyrosine Kinase 2**	NTRK2	Neurology	Inverse	[54]

Cardiometabolic, inflammatory, and neurology disease biomarkers associated with disease severity, according to the elastic net logistic regression analyses. Columns indicate, for each biomarker, its full name, its short name, whether a direct or inverse relationship between the biomarker level and disease severity was identified, and literature data supporting our findings. Biomarkers highlighted in grey have not yet been associated with severe COVID-19 by other authors. * an opposite, yet significant association was described in the literature. † literature results do not reach significance.

**Table 4:** Summary of biomarkers emerging from the correlation discovery analyses.

	Short Name	Model	Literature Data
**C-X3-C Motif Chemokine Ligand 1**	CX3CL1	ENLR	[54]
**CD200 Receptor 1**	CD200R1	ENLR, MI, GINI, SHAP, RFE	[54,57]
**Complement component 2**	C2	ENLR	[43]
**CUB domain-containing protein 1**	CDCP1	SHAP	[54]
**Dorsal Inhibitory Axon Guidance Protein**	DRAXIN	MI	[54]
**Ezrin**	EZR	MI, GINI, RFE	[54]
**Fetuin B**	FETUB	ENLR	[41]
**Glial Cell Derived Neurotrophic Factor Receptor α-3**	GDNFR-α 3	GINI	[54]
**Growth/Differentiation factor 8**	GDF8	MI	[54]
**Hepatocyte Growth Factor**	HGF	MI, GINI	[26,55]
**Insulin-like Growth Factor Binding Protein 3**	IGFBP3	ENLR	[40]
**Intercellular Adhesion Molecule 1**	ICAM1	MI	[52] †
**Interleukin 12**	IL12	ENLR	[52]
**Interleukin 12B**	IL12B	ENLR	[52]
**Interleukin 6**	IL6	ENLR, MI, GINI, SHAP, RFE	[16,51]
**Interleukin 8**	IL8	GINI, SHAP	[17,39,55]
**KIT proto-oncogene receptor tyrosine kinase**	KIT	MI, GINI, SHAP, RFE	
**Kynureninase**	KYNU	MI	[54]
**Latent-Transforming Growth Factor Beta-Binding Protein 2**	LTBP2	ENLR. MI, GINI, SHAP, RFE	[39,58]
**Leukemia Inhibitory Factor**	LIF	ENLR, GINI	[55]
**Matrilin 3**	MATN3	ENLR, MI, GINI, SHAP, RFE	[54]
**Monocyte Chemoattractant Protein 1**	MCP1	ENLR, MI, GINI, SHAP, RFE	[17,24]
**Monocyte Chemoattractant Protein 3**	MCP3	ENLR, MI, GINI, SHAP, RFE	[50]
**Netrin receptor**	UNC5C	ENLR	[54] †
**Neuronal Cell Adhesion Molecule**	NrCAM	ENLR	[58]
**Neurotrophic Receptor Tyrosine Kinase 2**	NTRK2	SHAP	[54]
**Neurotrophic Receptor Tyrosine Kinase 3**	NTRK3	ENLR	[54]
**Osteoprotegerin**	OPG	ENLR	[54]
**Stem Cell Factor**	SCF	GINI	[17]
**Tenascin R**	TNR	ENLR	
**Tissue inhibitor of metalloprotease 1**	TIMP-1	ENLR, MI, GINI	[38]
**Tumor necrosis factor ligand superfamily member 11**	TNFSF11 or TRANCE, RANKL, OPGL	ENLR, MI, GINI, SHAP, RFE	[54]
**Tumor Necrosis Factor Receptor Superfamily member 12A**	TNFRSF12A	ENLR, RFE	[56]
**α** **2-microglobulin receptor-associated protein**	α2-MRAP	MI, GINI, SHAP, RFE	[54]

Correlation discovery analyses results. Table summarizing biomarkers associated with disease severity in the analyses conducted on the complete dataset, including demographic, clinical, anamnestic, hematochemical, and immunometric parameters. The columns show in which of the five models the biomarker was associated with the severity of the disease and which literature data confirm our results. Biomarkers highlighted in grey have not yet been associated with severe COVID-19 by other authors. ENLR: elastic net logistic regression; MI: mutual information analysis; GINI: GINI index analysis; SHAP: Shapley additive explanations analysis; RFE: recursive feature extraction analysis. † the literature results do not reach significance.

## Data Availability

Raw data are provided in Supplementary Table S1.

## Supplementary Materials

The following supporting information can be downloaded at: https://www.mdpi.com/article/10.3390/ijms23169161/s1.
